# Quarantine

**Published:** 1878-10

**Authors:** 


### Editorial.

QUARANTINE.—We take great pleasure in publishing, as will be found below, resolutions passed by the Medical Society of South Carolina, and the communication of its Secretary, asking for them a place in our journal. We have, for two years past, labored to promote the objects sought in the resolutions passed by our brethren of the Palmetto State. Opinions touching the subject of quarantining against yellow fever, coming from scientific and closely observing physicians in a city like Charleston, where the disease prevails, and where it may be so thoroughly studied in all its relations, are worth more than all the theories that have ever been promulgated.

As the resolutions justly intimate, humanity demands the abrogation of rules and laws which, without compensating good to the public or individuals, embarrass commerce, interrupt mail facilities, and otherwise interfere with the dearest interests of social life. Quarantine laws against yellow fever do this and more. To their observance, the public have long been taught to look for security against the ravages of the disease, and this leads to the utter neglect of the only means of protection—sanitary measures at home.

It is due the public, that errors on this subject, for which the medical profession is to a large extent responsible, should be corrected by members of that profession. The facts accumulated by the late Dr. Arnold and Dr. LeHardy, of Savannah, and the eminent physicians of Charleston, and other places, should be made known to others than readers of medical journals; and while for our pages we seek practical facts and common-sense views on this subject, we hope other avenues may also be sought, through which to correct public opinion.

*Action of the Medical Society of South Carolina.*—At the regular monthly meeting of the Medical Society of South Carolina, held September 2d, 1878, the following resolutions, offered by Dr. R. A. Kinloch, were adopted and ordered to be published:

*Resolved*, That we witness with surprise and mortification the attempt, on the part of the citizens of many sections of our country, to institute a futile and oppressive system of *land quarantine* against yellow fever.

2*d*. That this system, originating, as we believe, with a panic-stricken people, and supported by the teachings of theorists, is inconsistent with the most generally received views as to the origin and propagation of the disease in question, and opposed to the humanity of a civilized age.

3*d*. That we respectfully urge upon the profession, throughout the length and breadth of our land, the necessity of opposing this false and inhuman doctrine by every means in their power, even, if necessary, by an earnest appeal for legislative enactments on the subject.

4*th*. That we respectfully, but most urgently, advise our fellow-citizens of those localities, when an invasion of the disease may seem imminent, to expend all their efforts rather in the removal of those causes, which, in accordance with the well-established facts of modern science, are *known to be potent in localizing epidemic disease.*

5*th*. That we extend our most heart-felt sympathy to our fellow-citizens who are now feeling the dire effects of the illegal and inhuman enactments referred to, and we pledge ourselves to do what we can in our own State, to aid in their deliverance, and to provide for their future security.

A true copy. W. H. Bailey, M.D.,
Sec. Med. Soc. S. C.

---

Charleston, September 15, 1878.

*To the Editors of the Atlanta Medical Journal:*

Gentlemen—In accordance with instructions from the Medical Society of South Carolina, the foregoing resolutions are forwarded to you with the request that they be inserted in the next number of your valuable journal. Similar copies have been furnished to other prominent medical journals of the South and West.

I am, very respectfully, your obedient serv't,

W. H. Bailey, M. D.,
Sec. Med. Soc. S. C.

